# Nitrogen deficiency regulates premature senescence by modulating flag leaf function, ROS homeostasis, and intercellular sugar concentration in rice during grain filling

**DOI:** 10.1186/s43141-021-00275-3

**Published:** 2021-11-23

**Authors:** Shamsu Ado Zakari, Syed Hassan Raza Zaidi, Mustapha Sunusi, Kabiru Dawaki Dauda

**Affiliations:** 1 Institute of Crop Science, College of Agriculture and Biotechnology, Zhejiang University, Hangzhou, 310058 China; 2 Department of Crop Production, Audu Bako College of Agriculture, Dambatta, Kano, Nigeria; 3 Department of Agronomy, University of Agriculture, Dera Ismail Khan, 29220 Pakistan; 4 Department of Crop Science, Faculty of Agriculture, Federal University Dutse, Jigawa, Nigeria; 5 Department of Crop Science, Kano University of Science and Technology, Wudil, Kano, Nigeria

**Keywords:** Nitrogen deficiency, Premature leaf senescence, Reactive oxygen species (ROS), C/N ratio, Carbon and nitrogen metabolites

## Abstract

**Background:**

Leaf senescence occurs in an age-dependent manner, but the rate and timing of leaf senescence may be influenced by various biotic and abiotic factors. In the course of stress, the function, composition, and different components of photosynthetic apparatus occur to be synthesized homogeneously or degraded paradoxically due to different senescence-related processes. Nitrogen (N) deficiency is one of the critical environmental factors that induce leaf senescence, and its incidence may curtail leaf photosynthetic function and markedly alter the genetic information of plants that might result in low grain yield. However, the physiological and genetic mechanism underlying N deficiency regulates premature senescence, and flag leaf function, ROS homeostasis, and intercellular sugar concentration in rice during grain filling are not well understood. In this paper, Zhehui7954 an excellent *indica* restorer line (wildtype) and its corresponding mutant (*psf*) with the premature senescence of flag leaves were used to study the effect of different N supplies in the alteration of physiological and biochemical components of flag leaf organ and its functions during grain filling.

**Results:**

The results showed that the *psf* mutant appeared to be more susceptible to the varying N supply levels than WT. For instance, the *psf* mutant showed considerably lower Pn, Chl *a*, Chl *b*, and Car contents than its WT. N deficiency (LN) decreased leaves photosynthetic activities, N metabolites, but significantly burst O_2_^•−^, H_2_O_2_, and relative conductivity (R1/R2) concentrations, which was consistent with the expression levels of senescence-associated genes. Sucrose, glucose, and C/N ratio concentrations increased with a decrease in N level, which was closely associated with N and non-structural carbohydrate translocation rates. Increases in POD activity were positively linked with the senescence-related enhancement of ROS generation under LN conditions, whereas, SOD, CAT, and APX activities showed opposite trends. High N (HN) supply significantly inhibits the transcripts of carbohydrate biosynthesis genes, while N assimilation gene transcripts gradually increased along with leaf senescence. The *psf* mutant had a relatively higher grain yield under HN treatment than LN, while WT had a higher grain yield under MN than HN and LN.

**Conclusions:**

This work revealed that the C/N ratio and ROS undergo a gradual increase driven by interlinking positive feedback, providing a physiological framework connecting the participation of sugars and N assimilation in the regulation of leaf senescence. These results could be useful for achieving a higher yield of rice production by appropriate N supply and plant senescence regulation.

**Supplementary Information:**

The online version contains supplementary material available at 10.1186/s43141-021-00275-3.

## Background

In cereal crops life cycle, leaf senescence is a critical developmental stage that eventually leads to self-destruction in the whole plant: program cell death [1]. Functionally, leaf senescence can be described based on a genetic program that regulates the degradation of the photosynthetic apparatus and the remobilization of metabolites to developing sink tissues [2]. Leaf senescence occurs in an age-dependent manner, but the rate and timing of leaf senescence may be influenced by various biotic and abiotic factors [3]. These factors rapidly weaken the photosynthetic components and increase excess reactive oxygen species (ROS) generation. Exposure of the plant to environmental adversities might alter the biochemical processes, which may disturb the equilibrium between the physiology and biochemistry in the plant life cycle [4]. In plant, the imbalance in biochemical components resulted in the exoneration of the inefficient and aging photosynthetic compounds that are essentially acquired by sink organs from the source.

Nitrogen (N) is an essential mineral element that affects plant photosynthetic ability, biomass production (growth and development), and grain yield. The status of N in plant leaves is closely associated with the photosynthetic period of functional leaves and also with the timing of leaf senescence [3, 5]. In cereals plant, N deficiency may lead to the low photosynthetic ability of leaf organs, stunted plant growth, low biomass production, and grain yield [3, 6]. Interestingly, literature reports suggested that the annual plants generally reserved up to 80% of the total N amount in the chloroplasts of photosynthetic organs, and the breakdown of chloroplasts allows leaf tissue to make its final contribution to other tissues by mobilizing the nutrients in the senescent leaves [7, 8], but during early senescence, the recycling of N from chloroplasts to other tissues requires the integrity of nuclei and cellular membrane, mitochondria [9]. However, most of these studies were conducted at seedling stages. Hence, a detailed understanding of the changes in biochemical contents with differential responses to N supply and its relation to premature senescence after anthesis in rice plants remained unclear.

Carbohydrate (C) and N availability is an important factor in the regulation of plant senescence [10, 11] and has a close relationship to crop yield; they are thus important current targets for improving nitrogen use efficiency (NUE), particularly for developing new cultivars. In *Arabidopsis*, growth analysis at different C/N ratios revealed that the C and N balance rather than N or C alone plays a significant role in stored lipid remobilization, seedling growth regulation, and photosynthetic gene expression [12]. Under stressful conditions, the inhibition of carbohydrate translocation or its export from the source organ (leaf) may result in an extra photosynthetic light energy, which may, in turn, lead to a burst of oxidative damage, such as the production of ROS [10, 13]. These oxidative stresses can easily cause the peroxidation of membrane lipids and speed up the senescence process. To cope with the oxidative stress effect, carbohydrates provide energy source and primary substance for the ROS scavenging systems [14]. However, the role of intercellular sugar concentration as part that induces leaf senescence has remained argumentative. Some authors found that low sugar concentration in plat resulted in reduction of photosynthesis activities, enhanced ROS production, and induced leaf senescence [15, 16]. However, other authors [17–19] gave data that were interpreted to underpin the opposite idea: leaf senescence is due to high sugar levels. Likewise, differences between natural and induced senescence studies unraveled many questions concerning senescence and sugar accumulation, but many other questions arise. In our previous study, we have confirmed that LN application enhanced soluble sugars and starch translocation due to weaker N assimilation and stronger C metabolism in leaf sheaths which might be closely associated with the initiation and progression of flag leaf senescence during grain [3]. However, it remains unclear how N deficiency affects sugar concentration in rice leaves by regulating the expression of these sugar isoforms and its relation to NSC translocation during leaf senescence.

Leaf senescence is regularly associated with increased oxidative damage on cellular macromolecules by ROS. Besides enzymatic and non-enzymatic ant-oxidative defense systems, many osmoprotectants involved in C and N metabolism are important in scavenging or detoxifying ROS and reducing lipid peroxidation [20]. Previously, we have reported that N deficiency significantly enhanced abscisic acid (ABA) concentration and ROS level in rice leaves, thus, attributable to the activation of ABA biosynthesis and also the suppression of ABA catabolism in rice leaves [21]. Hence, clarifying the involvement of different N supplies in leaf function, changes of biochemical components, and transcriptional expression of key genes involved in N assimilation and C allocation during senescence for speculating their functions in scavenging ROS production may be useful.

Grain filling is a necessary process that determines the ultimate yield of rice, after anthesis a substantial amount of N is required for grain formation, and leaf N dynamics might affect grain filling remarkably [22]. Leaf N translocation to the panicle is a prerequisite for the formation of good grains during grain filling. Grain filling and N recycling in rice plants are complex traits that depend on many factors such as rate of senescence to provide N source, photosynthesis to provide carbon skeleton, and re-utilization of precursors in sink organs for storage of protein and starch among others [23]. The early senescence of flag leaves may result in considerable loss of grain yield, quality, and rice production [23]. As both C allocation and N availability dictate grain quality, it is evident that understanding these relationships is essential in crop improvement and critical when developing novel cereal cultivars.

In this study, premature flag leaf senescence mutant rice (*psf*) and its wild type (WT) were employed with the sole aims of investigating and understanding senescence-related changes in biochemical components and leaf function of rice during grain filling under different N supplies. These results could be useful for understanding the regulatory mechanism of premature senescence, appropriate N supply under different conditions, and improving yield and quality traits in rice plants.

## Materials and methods

### Plant materials and nitrogen treatment

Two rice genotypes, Zhehui7954 an excellent *indica* restorer line (wildtype) and its corresponding mutant (*psf*) with the premature senescence (*psf*) of flag leaves, were used in this study. The *psf* mutant was developed from Zhehui7954 cultivar (*Oryza sativa* L. ssp. *indica*) mature seeds with gamma irradiation as a mutant factor, and the stably inherited mutant was obtained through successive self-pollination for more than eight generations. The hydroponic N supply experiments were performed at the experiment station of Zijingang campus (30° 18N, 120° 04E) Zhejiang University, Hangzhou, China. Rice seeds were sown on seedling beds after indoor pre-germination. The uniform 30-day-old seedlings were subsequently transplanted to plastic pots filled with 3.5 L standard IRRI (International Rice Research Institute) nutrient solution, and four rice seedlings were transplanted for each pot. The pots were positioned in a greenhouse under natural light conditions and temperatures of 28 °C day/22 °C night. After 30 days of normal growth in the IRRI nutrient solution, all rice plants were randomly divided into three groups (nine pots for each group) and then subjected to different N supply treatments until rice maturity.

Three N levels in the hydroponic culture were designed: 1.45 mM (low N level; LN), 2.90 mM (medium (N level; MN) (As control), and 5.80 mM (high N level), respectively. Ammonium nitrate (NH_4_NO_3_) was used as a source of N. The MN (control) treatment (2.90 mM) is according to the standard formulation in IRRI protocol, with the 1/2 and 2× levels of standard N concentration in IRRI solution being set for LN and HN, respectively. The concentration of all other mineral elements was the same except N element. The solution in the plastic pots was renewed every 6 days, and pH was adjusted to 5.7−5.8. The flag leaves of rice plants were sampled at the grain-filling stage. At the full heading stage of rice plants for each N treatment, the rice panicles with uniform anthesis day were tagged in each pot and subsequently sampled at an interval of 15 days after anthesis. The flag leaf of the tagged panicles in rice plants (three pots) was used for each sampling time. Sampled flag leaves samples were immediately frozen in liquid nitrogen and kept at − 80 °C until further experimental analysis portion of the sampled leaf was dried to constant weight in an oven and ground into fine powder for the measurement of sugars and N content. At grain maturity, the available panicle per plant, number of grains per panicle, grain weight, and seed-setting rate were measured to estimate the grain yield of rice plants by using another three pots (without the flag leaf being sampled) for each N treatment.

### Measurement of photosynthesis activities

The net photosynthesis rate (Pn) using an LI-6400 portable photosynthesis system (Li-Cor Inc. USA) and the Chl content was assayed spectrophotometrically using a Shimadzu UV-vis 2450/2550 spectrophotometer (Shimadzu, Japan) while chlorophyll fluorescence was determined using a portable chlorophyll fluorometer PAM-2000 (WALZ, Germany) as previously described in [3, 21].

### Transmission electron microscopy (TEM)

The fragments from the fully expanded flag leaves, without midrib, were collected from randomly selected plants and fixed overnight in 4% glutaraldehyde (v/v) in 0.1 mol L^–1^ sodium phosphate buffer (PBS) (pH 7.4), washed three times with the same buffer. The samples were fixed in 1% osmium tetraoxide (OsO_4_) for 1 h and washed three times for 10-min intervals between each washing. Then the samples were dehydrated in a graded series of ethanol and successively washed with absolute acetone for 20 min. The specimens were then infiltrated and embedded in Spurr’s resin overnight, then heated at 70 °C for 9 h. The ultra-thin sections specimens (80 nm) were mounted on copper grids for viewing by a transmission electron microscope (JEOL TEM-1230EX) at an accelerating voltage of 60.0 kV [6].

### Determination of hydrogen peroxide (H_2_O_2_), superoxide radical (O^2•−^), and relative conductivity (R1/R2)

H_2_O_2_, O^2**•−**^ concentrations, and R1/R2 were determined as described previously by [24, 25], respectively. Triplicate measurements were conducted for each sample.

### Histochemical staining

The H_2_O_2_ concentration is qualitatively estimated using 3,3-diaminobenzidine (DAB) as previously described by [21] employing the method of [26]. Flag leaf sections were excised to detect O^2**•−**^ accumulation by a 0.1% solution of NBT in 10 mM potassium phosphate buffer (pH 7.8), according to [27]. After incubation, the leaves were set in 2:1:1, 95% ethanol:lactic acid:phenol (alcoholic lactophenol), kept at 65 °C for 30 min, rinsed with 50% ethanol, and then rinsed with water. A blue precipitate form is visible in leaves when NBT interacts with O^2**•−**^. The pictures were taken by using a digital camera.

### Determinations of antioxidant enzymes activities

The antioxidant enzymes peroxidase (POD; EC 1.11.1.7), catalase (CAT; EC 1.11.1.6), superoxide dismutase (SOD; EC 1.15.1.1), and ascorbate peroxidase (APX; EC 1.11.1.11) activities were assayed and measured spectrophotometrically (Shimadzu UVevis 2450/2550, Shimadzu, Japan) according to the procedure described in [24]. Triplicate measurements were assayed for each sample.

### Determination of carbohydrate and N metabolites

The sugars extraction and concentration measurement was performed by following the method of [28, 29], respectively. Starch content was determined as previously described in [3] using the procedure of [30]. The total N contents were measured using an Elemental analyzer (Elementar) while soluble protein content was determined in fresh leaves by the method of [31].

### RNA isolation, cDNA preparation, and quantitative real-time PCR (qRT-PCR)

The RNA extraction and cDNA preparation for flag leaf tissues were performed as described by [24]. Total RNA was extracted by using TRIzol reagent (Invitrogen, Carlsbad, CA, USA); the ReverTra Ace qPCR RT Kit (Toyobo, Osaka, Japan) was used for cDNA synthesis following the manufacturer’s instructions. The qRT-PCR was performed as described in [21]. All gene-specific primer pairs used in this study are listed in Table S1. The Actin (X16280) gene was used as an internal control. The 2^(-ΔΔCT)^ method was used to determine the relative expression levels of the various genes used in these experiments.

### Statistical analysis

Statistical differences were obtained by means of the analysis of variance with SPSS v. 18.0 (IBM Corp., Armonk, NY, USA). Means were compared by least significant difference (LSD) test (*p*<0.05). The standard deviation (SD) was calculated and shown in figures and tables.

## Results

### Senescence-associated changes in photosynthesis activities, carbon and nitrogen metabolites, transcriptional expression of key genes involved in N assimilation, and C allocation in the flag leaves under different N supplies

A distinct difference in photosynthesis activities and chlorophyll fluorescence was observed between different N supplies and rice cultivars (Table 1). Under LN treatment, the photosynthesis activities SPAD value, Chl a, b, and Pn in flag leaves were significantly lower than those under MN and HN for the same sampling period(s). However, Chl b was degraded faster than Chl a during senescence in the rice leaves. There was a marked difference in the temporal pattern of Fm/Fo and Fv/Fm ratios; the Fm/Fo ratio was significantly lower under N deficiency and continuously declined (LN) from heading (0 DAA) to harvesting period. These values were basically similar to the temporal pattern in the genotypic differences; *psf* mutant differs significantly from its WT with a substantial decrease in terms of photosynthesis parameters measured. LN induced leaf senescence earlier in the *psf* mutant than the WT, while HN showed an inhibitory effect.

**Table 1:** Differences in the senescent-associated parameters in the flag leaves of the *psf* mutant and its wildtype under different N supply levels

Sampling stage	Genotype (G)	N Level (N)	Chl (a) (mg g^−1^ FW)	Chl (b) (mg g^−1^ FW)	SPAD	Pn (μmol CO_2_ m^−2^ s^−1^)	Fv/Fm	Fm/Fo
0 DAA	Wildtype	HN	4.09±0.43a	1.89±0.19a	37.76±2.30a	17.1±1.84a	0.82±0.012a	5.54±0.20a
		MN	3.82±0.22b	1.82±0.12a	35.61±3.10a	11.1±0.08b	0.71±0.10b	5.13±0.41a
		LN	2.03±0.21c	1.26±0.18b	23.44±2.37b	10.3±0.08c	0.51±0.014c	3.51±0.24b
		Means	3.45A	1.59A	32.27A	12.85A	0.68A	4.73A
	*psf*	HN	4.19±0.34a	1.50±0.12a	35.70±1.26a	11.9±0.18b	0.72±0.014a	5.01±0.21a
		MN	3.61±0.18b	1.61±0.23a	30.72±1.53b	15.3±0.10a	0.57±0.03b	3.57±1.00b
		LN	1.29±0.33c	1.00±0.22b	21.01±0.98c	9.6±0.35c	0.44±0.012c	2.33±0.27c
		Means	3.03AB	1.37A	29.14B	12.31A	0.57B	3.64B
15 DAA	Wildtype	HN	3.64±0.13a	1.38±0.05a	35.41±0.35a	15.4±0.33a	0.86±0.036a	4.63±0.13a
		MN	2.05±0.25b	1.09±0.10ab	32.26±0.20a	10.0±0.38b	0.64±0.031b	3.01±0.41b
		LN	1.69±0.22c	0.92±0.05b	19.83±0.30b	5.6±0.03c	0.36±0.009c	2.36±0.09c
		Means	2.469A	1.13A	29.16A	10.39A	0.62A	3.33A
	*psf*	HN	3.71±0.36a	1.13±0.12b	30.87±1.17a	8.9±1.78a	0.65±0.014a	3.83±0.11a
		MN	2.16±0.31b	1.56±0.26a	21.68±2.17b	7.5±0.82b	0.42±0.03b	2.80±0.54b
		LN	0.64±0.06c	0.50±0.11c	14.51±1.15c	3.23±0.78c	0.28±0.003c	2.02±0.03c
		Means	2.17AB	1.06A	22.35B	6.57B	0.45B	2.88AB
Harvesting	Wildtype	HN	2.55±0.27a	1.29±0.12a	23.82±1.36a	10.2±1.88a	0.56±0.027a	4.02±0.17a
		MN	1.81±0.06b	1.01±0.08ab	18.11±0.20b	5.0±0.69b	0.44±0.03b	2.97±0.61b
		LN	1.18±0.11c	0.86±0.11b	10.80±1.00c	1.4±0.29c	0.36±0.010c	1.5±0.01c
		Means	1.85A	1.05A	17.57A	5.57A	0.45A	2.83A
	*psf*	HN	1.31±0.58a	0.81±0.19a	20.04±3.10a	4.7±0.48a	0.39±0.016a	2.22±0.01a
		MN	1.17±0.08a	0.74±0.24a	11.01±1.02b	4.0±0.81b	0.21±0.001b	1.01±0.10b
		LN	0.25±0.12b	0.24±0.04b	8.47±1.02c	1.1±0.36c	0.17±0.005	0.72±0.05c
		Means	0.91B	0.60B	13.17B	3.30B	0.25B	1.32B
Means effect		Nitrogen	*	*	*	**	**	*
		Genotype	*	ns	*	*	*	*
Interaction		N*G	ns	ns	*	*	*	ns

Different letters within the columns indicate a significant difference between nitrogen treatments for a genotype and mean values indicate a significant difference between the genotypes under all the nitrogen levels at the same sampling stage at *p* ≤ 0.05 using the least significant difference (LSD)

*DAA* days after anthesis, *HN* high N, *MN* medium N, *LN* low N

** and * indicate the significance at the 0.01 and 0.05 level, respectively; NS means no significant difference based on the analysis of variance. Data are presented as means ± SD (*n*=3)

**Fig. 1:**
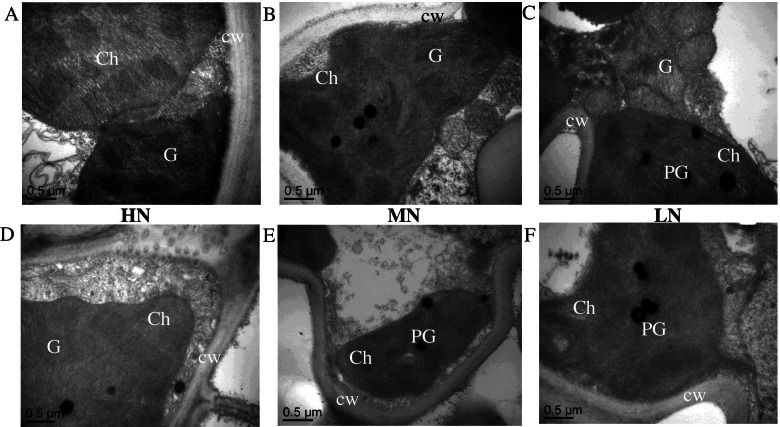
Effect of different nitrogen supplies on chloroplast ultrastructure between wildtype (**A**–**C**) and *psf* mutant (**D**–**F**). Chloroplast structure and thylakoid organization in flag leave at 15 days after anthesis were analyzed by transmission electron microscopy (TEM). **A**, **D** HN; **B**, **E** MN; and **C**, **F** LN

**Fig. 2:**
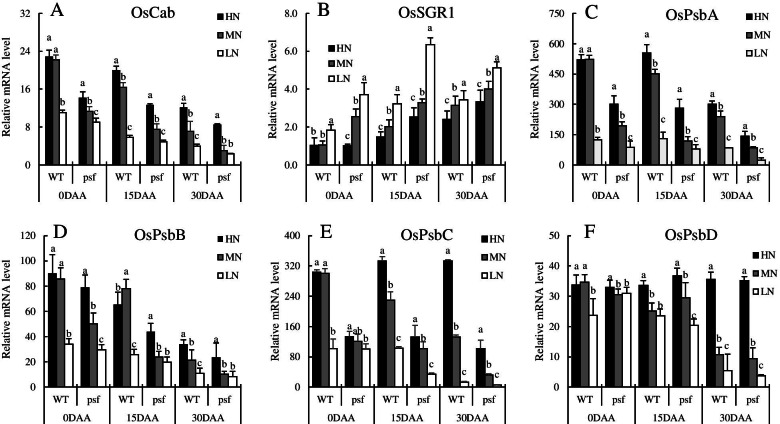
Differential expression level of **A** gene encoding light-harvesting chlorophyll a/b binding protein (Cab), **B** Chl degradation associated gene (OsSGR), **C** photosystem II binding proteins A (*OsPsbA*), **D** (*OsPsbB*), **E** (*OsPsbC*), and **F** (*OsPsbD*) in the flag leaves of wildtype and psf mutant under different N levels during leaf senescence process. Relative gene expression levels were normalized to those of actin at corresponding time points. Data are presented as means ± SD (n=3). Different letters indicate significant differences at P < 0.05 (LSD) between nitrogen treatments, respectively

The ultrastructure of the chloroplast in the flag leaf at 15 DAA was observed through transmission electron microscopy (Fig. 1). Under HN, the chloroplasts were predominantly ellipsoid in shape, and the cell wall, stromal lamellae, and grana were well developed, as well as a fewer number of plastoglobuli, while thylakoids were assembled densely beside the long axis of the chloroplasts (Fig. 1A, D). While under LN, structural deformation of chloroplast alongside de-stacking of thylakoid and stromal lamellae was quite apparent. The inner membrane system was composed of a lower number of grana containing fewer thylakoids (Fig. 1C, F). The chloroplasts of *psf* mutant showed an increased number of plastoglobuli and stroma thylakoids with ruptured membranes (Fig. 1D–F). From these results, it can be infrared that LN strikingly decreased photosynthesis machinery during grain filling and played a prominent role in the initiation and progression of leaf senescence. To confirm this tendency, we measured mRNA expression levels of genes associated with encoding light-harvesting chlorophyll a/b binding protein and Chl degradation, respectively. Interestingly, HN had a significantly higher transcript amount of *OsCab* than MN and LN (Fig. 2A), the expression level decreases with progression of leaf senescence, while the opposite was true for *OsSGR1* (Fig. 2B), thus consistent with Chl content. Additionally, the expression level of essential genes encoding for the reaction center of the photosystem II (PSII) complex was measured. As shown in Fig. 2C–F, the LN condition inhibits the expression levels of *OsPsbA*, *OsPsbB*, *OsPsbC*, and *OsPsbD* in comparison to the other two N treatments. Clarifying that, N deficiency weakened energy transfer and reduced electron transport ability of light-harvesting through the reaction center of PSII. For the genotypic differences, the expression level of genes encoding for the PSII in the *psf* mutant rice leaves was significantly lower than those of the WT and decreased gradually during the entire sampling period, indicating that the premature leaf senescence of the *psf* mutant may be characterized by the distinctly decreasing electron transfer efficiency in PSII reaction center and critically suppressed its activity, as denoted by a severe lessening in Fv/Fm and Fm/Fo for the senescing flag leaves of the *psf* mutant.

**Fig. 3:**
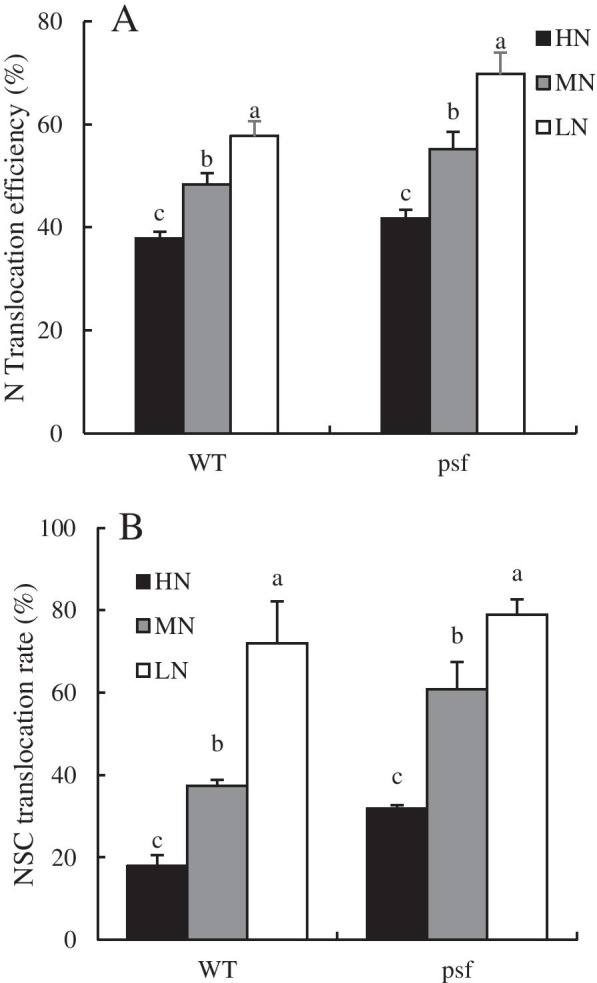
Carbon and nitrogen translocation rate in the flag leaves of psf mutant and its wildtype under different nitrogen supply. Data are means ± SD (n=3). Different letters indicate a significant difference between N treatments at p < 0.05 least significant difference (LSD). N translocation efficiency (NTE) = (nitrogen translocation/leaf N content at anthesis) × 100. NSC translocation rate = [NSCs in leaf at the heading stage − NSCs residue of the leaf at the harvest stage]/NSCs in leaf at the heading stage × 100

The intercellular sugar levels were considered as part of the pathway that regulate leaf senescence, thus, we measured the carbohydrate concentrations in the flag leaves (Table 2). The effect of N deficiency on sugars varies with the sampling period. For instance, LN significantly enhanced fructose and NSC at the initial stage of leaf senescence (0 DAA), but it caused a considerable decrease in their concentration at the middle (15 DAA) and late stages of leaf senescence, while sucrose showed an opposite trend. However, glucose content and C/N ratio increases with decreases in N supply, an opposite trend was observed in soluble protein and leaf N contents. The decreases in those cellular metabolic indicators are relatively higher in the flag leaf of *psf* mutant than the WT. As an objective way to quantify N and NSC translocations from leaves during senescence. We measured N-translocation efficiency (NTE) and NSC translocation rate (Fig. 3). Both NTE and NSC translocation decrease with the increase in N supply, and the translocations efficiency was delayed in the flag leaves of WT compared to the *psf* mutant under the same N conditions (Fig. 3A, B).

**Table 2:** Differences in carbon and nitrogen metabolites in rice flag leave with differential response to nitrogen level

Sampling period	Genotype (G)	N Level (N)	Sucrose (mg g^−1^)	Fructose (mg g^−1^)	Glucose (mg g^−1^)	NSC (%)	Soluble protein	Leaf N content (%)	C/N ratio
0 DAA	Wildtype	HN	53.04±2.30a	56.31±2.25b	22.17±5.21a	16.39±2.07b	18.40±1.58a	5.84±0.54a	1.64±0.17ab
		MN	48.35±4.20a	56.30±1.00b	22.23±4.58b	16.25±1.04b	15.37±2.01b	4.96±0.31ab	1.77±0.21ab
		LN	42.47±4.13b	89.80±1.06a	30.99±5.36c	19.23±1.31a	10.76±2.23c	2.51±0.04b	2.10±0.24a
		Means	47.95B	67.14B	25.13B	16.834B	14.84A	4.43A	1.82ns
	*psf*	HN	60.17±3.78a	56.77±10.65b	27.41±6.13a	17.66±3.10c	17.17±1.11a	5.68±0.13a	1.57±0.35ab
		MN	52.32±3.01b	61.06±8.05b	32.64±2.85b	18.73±2.10b	17.32±2.21b	3.81±0.51b	2.11±0.21a
		LN	45.86±2.14c	95.97±12.86a	40.11±2.38c	20.78±2.05a	8.99±1.62c	2.02±0.008c	2.29±0.51a
		Means	52.78A	71.27A	33.38A	18.286A	14.49A	3.83AB	1.76ns
15 DAA	Wildtype	HN	30.70±4.51c	55.75±10.18c	20.67±3.09a	15.12±2.16a	15.17±4.01a	5.01±0.2a	1.66±0.21ab
		MN	34.03±1.08b	49.61±5.90b	20.21±3.21b	14.18±1.90ab	11.30±1.07b	3.86±0.1b	2.08±0.21a
		LN	45.77±1.60a	36.69±8.05a	25.61±2.41c	10.73±0.83b	5.41±0.91c	2.33±0.12c	2.39±0.32a
		Means	36.83B	47.35B	22.16B	13.21A	10.62B	3.73A	1.92ns
	*psf*	HN	40.20±1.73c	60.89±7.75a	22.54±7.03a	15.33±1.72a	16.97±1.60a	4.30±0.52a	1.77±0.48a
		MN	45.51±2.00b	55.02±12.25b	28.86±3.52b	11.10±2.38b	14.61±1.24b	3.19±0.8b	1.88±0.18a
		LN	48.32±2.71a	48.72±5.73c	32.90±1.42c	6.69±1.13c	8.98±1.18c	1.50±0.01c	2.01±0.27a
		Means	44.67A	54.88A	28.1A	11.04B	13.52A	3.00AB	1.85ns
30 DAA	Wildtype	HN	23.31±1.36b	50.19±10.14a	14.37±3.15a	13.44±1.91a	15.28±2.41a	3.63±0.04a	2.27±0.30b
		MN	25.44±2.32b	30.58±3.65b	18.69±1.20b	10.16±2.01b	9.72±1.00b	2.68±0.11b	2.29±0.34b
		LN	30.05±1.06a	15.12±6.02c	20.18±1.9bc	5.38±0.41c	6.10±0.37c	1.06±0.04c	3.17±0.19a
		Means	26.26B	31.96A	17.74B	9.81A	10.36A	2.457A	2.472A
	*psf*	HN	24.84±2.59c	31.58±7.86a	18.6±2.6a	12.03±2.19a	11.50±1.36a	2.82±0.02a	2.30±0.51b
		MN	30.03±2.01b	25.33±2.05b	22.36±2.84b	7.33±0.94b	7.32±0.71b	1.80±0.01b	2.70±0.34b
		LN	33.69±0.94a	14.97±1.45c	23.77±1.03c	4.39±0.64c	2.21±0.07c	0.81±0.04c	3.55±0.37a
		Means	29.52A	23.96B	21.57A	6.447B	7.01B	1.81B	1.80B
Means effect		Nitrogen	*	**	**	*	*	*	ns
		Genotype	*	*	**	*	ns	*	ns
Interaction		N*G	ns	*	*	ns	ns	*	ns

Different letters within the columns indicate a significant difference between nitrogen treatments for a genotype and mean values indicate a significant difference between the genotypes under all the treatments at *p* ≤ 0.05 level least significant difference (LSD)

*DAA* days after anthesis, *HN* high N, *MN* medium N, *LN* low N

** and * indicate the significance at the 0.01 and 0.05 level, respectively; NS means no significant difference based on the analysis of variance. Data are presented as means ± SD (*n*=3)

**Fig. 4:**
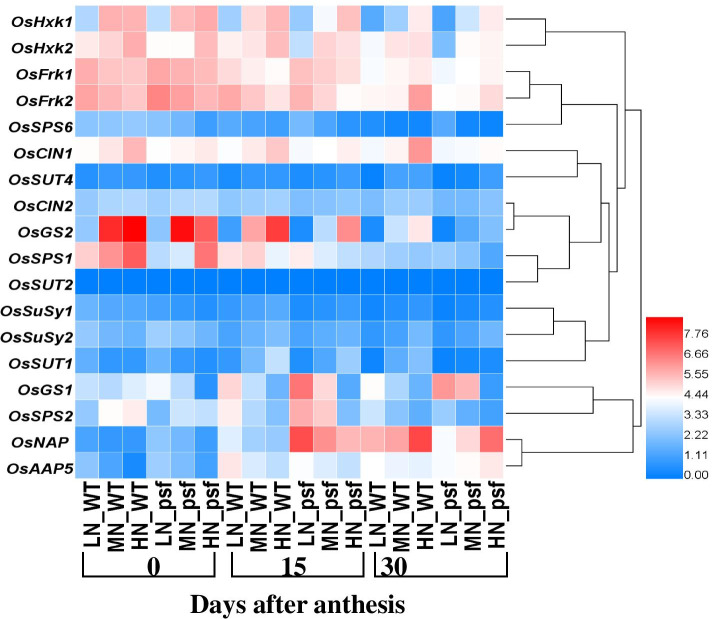
Expression profiles and temporal pattern of some key genes related to carbon and nitrogen assimilation as heat map; the expression of 18 genes was a profile in the flag leaves of *psf* and wildtype imposed to different N levels, at different three stages of grain filling

To confirm these differences, we measured the relative mRNA levels of *OsNAP* and *OsAAP*: those genes related to N translocation. LN supply induced transcripts level of both *OsNAP* and *OsAAP* significantly, while HN inhibit their transcript amounts (Fig. 4), thus consistent with a lower NTE. To understand the relationship of C and N assimilation in rice leaves with the initiation and subsequent progression of leaf senescence, the transcriptional expressions and temporal pattern of key genes involved in a limiting rate step of sugar and N acclimatization in response to different N levels were comprehensively investigated by qRT-PCR (Fig. 4). Remarkably, HN significantly enhanced the transcriptional expression of *OsGS2* at an early stage (0DAA) and gradually decreased along with leaf senescence (Fig. 4), but the transcriptional expression of *OsGS1* showed an opposite trend. Considering the remarkably low transcript amount of N assimilation genes in rice leaves under N deficiency, we inferred that the senescence-related changes in *psf* mutant rice leaves were mostly caused by the imbalance of N biosynthesis and also Chl degradation. On the other hand, HN supply suppressed the expression of *OsGS1* (at the initial stage) genes in N biosynthesis pathway. Hence, HN had an inhibitory effect on assimilation in leaf tissues, which in turn resulted in the delayed leaf senescence.

Also, to characterize the conversion of sucrose and other monosaccharides in flag leaf at the molecular level, the transcription analysis of *CIN*, *FrK*, and *HxK* isoform genes was measured (Fig. 4). *OsCIN1* was preferentially expressed under HN treatment conditions, but the *OsCIN2* transcript was detected in an extremely low level.

*OsFrk1* and *OsFrk2* isoform genes transcript was the most abundant in flag leaf compared to other C conversion-associated isoforms; *OsFrk* 1 and 2 was upregulated under LN condition at 0 DAA, then gradually downregulated with aging. *OsHxk* isoform genes (1 and 2) showed a similar expression pattern with *OsFrk* isogenes (Fig. 4). The temporal transcripts of *OsCIN* and *OsHxk* isoforms in the flag leaf of *psf* mutant rice maintained a lower level than those in the WT cultivar (Fig. 4). However, *OsFrk* 1 and 2 were observed to be highly expressed in the flag leaf of *psf* mutant than those in WT during the early stages of anthesis, then declined progressively to a lesser level than those in the WT (Fig. 4). These results suggested that the conversion between sucrose and other nonstructural carbohydrates during senescence in rice flag leaf chiefly occurred in the initial stage of grain filling, which was modulated by the transcription of *OsCIN1*, *OsFrk1*, and *OsFrk2*. Expression of *OsSPS* isogenes varied among N levels and across developmental stages in general, and expression decreased in the order *OsSPS1*>*OsSPS2*>*OsSPS6*. However, the transcript of expression of *OsSUT1* appeared to be rhythmic under HN and MN, with the highest values observed at 15 DAA, whereas expression under LN was generally declined from heading to 30 DAA to a lower level than MN and HN; however, a similar trend was observed for *OsSUT4*. *OsSUT1* was highly expressed in leaf tissue, whereas *OsSUT2* was detected with shallow expression, but no transcripts were detected for *OsSUT3* and *OsSUT5* (Fig. 4). Similarly, *OsSuSy1* transcript decreases continuously with virtually no detectable levels observed at 30 DAA. Taken together, these data indicated how C and N assimilation reductions differ under sufficient and deficient N conditions with differential responses to cultivars also. Consequently, this phenomenon illustrates senescence phenotypes during the grain-filling period.

### Participation of ROS generation and antioxidant activities in N deficiency-induced leaf senescence

**Fig. 5:**
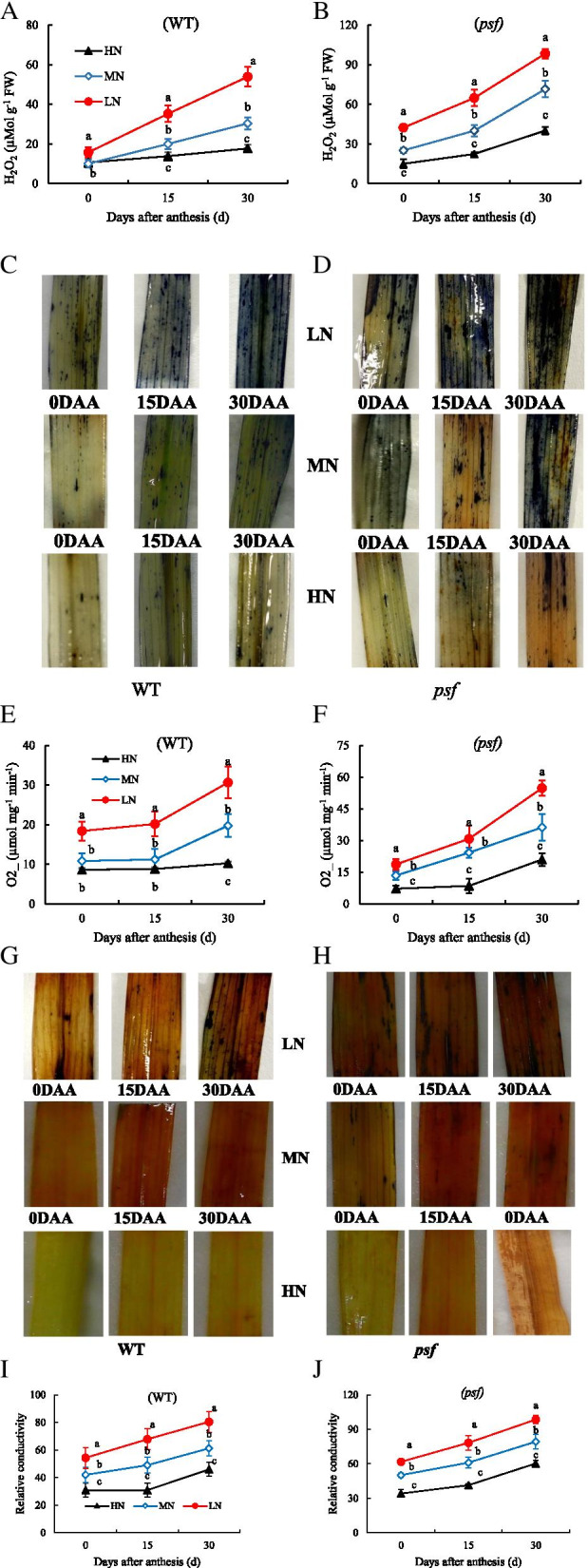
Impact of N deficiency/overfeeding on ROS bust and membrane integrity in the flag leaves of wildtype and *psf* mutant during senescence: **A**, **B** H_2_O_2_ accumulation, **C**, **D** NBT staining, **E**, **F** O_2_ concentration, **G**, **H** DAB staining, and **I**, **J** relative conductivity. Data are means of ±SD (*n*=3), different letters indicate a significant difference between treatments at a particular sampling period (*P* < 0.05) LSD

N deficiency significantly increased the degree of H_2_O_2_ and O_2_^**•−**^ generation and accumulation, consistently with the progression of leaves senescence and also more striking increases in R1/R2 level (Fig. 5). The *psf* mutant appeared to be more susceptible to the varying N supply levels than its WT, in terms of the varying extent of O_2_^**•−**^ and H_2_O_2_ production and also their production rates during senescence. Additionally, the marked increase in ROS production for the *psf* mutant tended to be concomitant with the rapid increase in relative conductivity level. By distinction, the H_2_O_2_ generation was markedly higher than O_2_^**•−**^ production (Fig. 5A–G). This result unambiguously showed that LN triggered the rapid induction of O_2_^**•−**^ and H_2_O_2_ generation, which accelerate leaf senescence, while HN resulted in a significant inhibition of ROS production and relative conductivity. The activities of antioxidant enzymes were further examined to compare the differences in scavenging the activities of ROS associated with senescence initiation and progression between the two genotypes under different N supplies (Table 2).

It showed that LN resulted in lower activities of SOD, CAT, and APX, but higher POD activity than MN and HN treatments. Moreover, the activities of SOD, CAT, and APX decrease with the progression of leaf senescence, but POD activity increases with the development of leaf senescence. However, the *psf* mutant differed obviously from its WT in the activities of these antioxidant enzymes at the subsequent phases of leaf senescence: SOD, CAT, and APX activities in the *psf* leaves deteriorated rapidly from heading day to maturity, whereas those in the WT leaves decreases gradually (Table 3). Interestingly, the POD activity in the *psf* mutant leaves increases promptly from heading to maturity days. These imply that the reduction in SOD, CAT, and APX in activities for N deficient leaves were closely related with the significant and persistent boost in H_2_O_2_ and O_2_^**•−**^ concentrations and were absolutely responsible for the ROS accumulation in the senescent leaves, considering the weak ability of LN to scavenge ROS in the senescing leaf.

**Table 3:** Difference in antioxidants enzyme activities in the flag leaves of *psf* mutant and its wildtype under different nitrogen supplies during grain filling

DAA	Genotype	N level	SOD activity (U mg^−1^ FW)	POD (U mg^−1^ FW)	CAT (μmol min^−1^ mg^−1^ FW)	APX (μmol min^−1^ mg^−1^ FW)
0	WT	HN	177.09±2.17a	23.20±1.94b	223.01±4.83a	36.95±2.30a
		MN	170.34±5.12b	31.34±5.06a	221.72±13.41b	38.01±5.00a
		LN	98.68±3.80c	33.64±1.71a	181.05±5.98c	28.35±1.07b
		Means	148.70B	29.39B	208.59A	34.44A
	*psf*	HN	277.83±3.72a	24.43±1.17c	251.83±3.29a	31.80±2.59a
		MN	210.08±10.22b	33.26±4.98b	244.21±10.23b	30.75±3.15a
		LN	88.96±5.24c	36.19±0.39a	103.54±2.39c	21.00±1.99b
		Means	192.29A	31.29A	199.86B	27.85B
15	WT	HN	127.63±13.18a	35.44±3.06c	274.92±12.30a	25.58±1.42a
		MN	105.74±5.87b	38.17±2.41b	181.00±6.89b	20.12±4.08b
		LN	53.43±3.11c	41.12±1.18a	138.18±14.14c	14.52±2.87c
		Means	95.60A	38.24B	198.03A	20.07A
	*psf*	HN	106.18±10.76a	43.92±2.97c	188.83±10.62a	21.21±0.46a
		MN	71.63±4.98b	49.00±5.01b	124.37±9.81b	18.04±2.05b
		LN	37.04±1.59c	54.45±2.22a	84.27±4.19c	11.32±1.17c
		Means	71.62B	49.12A	132.49B	16.86B
30	WT	HN	85.62±8.84a	40.99±3.12c	202.56±15.79a	13.62±1.39a
		MN	51.62±3.21b	46.21±3.14b	155.28±11.37b	10.23±1.25b
		LN	28.24±1.74c	50.10±1.74c	82.09±13.78c	9.58±1.01c
		Means	55.16A	45.77B	146.64A	11.14A
	*psf*	HN	53.48±4.06a	54.70±1.21c	91.13±16.00a	10.71±0.12a
		MN	30.02±6.14b	63.10±8.14b	72.51±6.77b	7.82±1.22b
		LN	21.81±2.01c	72.26±0.85a	21.72±7.22c	5.61±0.81c
		Means	35.10B	63.35A	61.79B	8.05AB
Means effect		Nitrogen	**	**	**	*
		Genotype	**	*	*	*
Interaction		N*G	*	*	*	ns

Different letters within the columns indicate a significant difference between nitrogen treatments for a genotype and mean values indicate a significant difference between the genotypes under all the treatments at *p* ≤ 0.05 level least significant difference (LSD)

*DAA* days after anthesis, *HN* high N, *MN* medium N, *LN* low N

** and * indicate the significance at the 0.01 and 0.05 level, respectively; NS means no significant difference based on the analysis of variance. Data are presented as means ± SD (*n*=3)

### Impact of different N supplies on grain yield traits and NSC contribution

There were significant differences in grain yield traits and also in the NSC contribution among the three N treatments (Table S2). LN significantly decreased available panicle per plant, the number of grains per panicle, grain weight, and seed-setting rate. Hence, N deficiency resulted in significantly lower grain yield than MN and HN. Interestingly, N reducing rate under LN were significantly higher than that under MN and HN treatments, and the order of different N treatments are as follows: LN>MN>HN, regardless of rice genotypes. Correspondingly, N deficiency led to a notably higher NSC contribution to grain than MN and HN; the NSC contribution to grain was 0.37% for WT and 1.44% for *psf* mutant. The effect of N supply on grain yield was somewhat variable, depending on rice genotypes. For the *psf* mutant, HN treatment had a relatively higher grain yield than the MN level, while for WT, the opposite trend was observed, implying that the extent of HN inducible decline in the carbon translocation rate from flag leaf to filling grain also was significantly varied among different rice genotypes. Considering the lower grain yield, higher reducing N rate, and NSC contribution of the flag leaf under N deficiency, we deduced that the positive effect from the significant enhancement in N and NSC translocation rate in flag leaves induced by N deficiency was not enough to balance the adverse effect of N deficiency on leaf photosynthetic ability and assimilation supply. Those could be reflected by the lower Chl and Pn in flag leaves under N deficiency, concomitantly with the occurrence of accelerated leaf senescence and more severe senescence symptom induced by LN at the middle and late stage of grain filling.

## Discussion

The occurrence of early leaf senescence caused by adverse stresses and intrinsic genetic limits the supply of photo-assimilates from the source leaves, thus, reduced photosynthetic activity [7]. The most prominent characteristic in the premature senescent leaf is the yellowing phenotype due to Chl degradation during chloroplast decomposition, while abundant Chl in the source leaf is required for the biosynthesis of photosynthesis [24]. Kumari [32] suggested that low N supply decreased the Chl fluorescence of primary leaves in sunflower and dissipated the excess energy under high irradiance. In the present study, N deficiency significantly decreased and instigated photosynthesis machineries and chlorophyll fluorescence, respectively. The decrease in the SPAD and Pn due to N deficiency-induced leaf senescence was likely the principal reason that led to the loss of final yield. Since the *psf* mutant was more susceptible to the effect of N deficiency, hence, the WT has more efficient photosynthesis activity. The decreases in Fv/Fm and Fm/Fo in *psf* mutant flag leaves during the entire sampling period explained the weakened abilities of light energy transfer and harvesting in mesophyll cells of senescent leaves. These effects were due to the low Chl a; responsible for energy transfer and excitation in photosynthesis and Chl b controlling light energy capture, an antenna chlorophyll, and energy assembly [24]. The degradation of Chl b was more complex than that of Chl a because the degradation of Chl b must first be converted to Chl a via 7-hydroxymethyl Chl a and possibly more sensitive to ROS that accumulated in mesophyll cells during leaf senescence [33]. Several lines of study have demonstrated that Chl takes a fundamental role in photosynthesis by f building complex with thylakoid-membrane proteins such as cytochrome b6f complex and photosystems I and II [33, 34]. In this study, the N deficiency condition and *psf* mutant rice showed a significant reduction in Fv/Fm value during the grain-filling period. The expression levels of genes encoding for the core reaction center of PSII complex (PsbA, PsbB, PsbC, and PsbD) were significantly lower for the *psf* mutant than those for the WT, concurrently with LN supply during the entire sampling period (Fig. 2C–F). Thus, signifying that the structure of the PSII complexes lost some biological function and thylakoid membrane was severely damaged in senescent leaves, as observed in senescent leaves via TEM (Fig. 1). However, the significantly decreased expression of Cab in senescent leaves (Fig. 2A) suggested the weakened harvesting of transfer of light energy and quantum photon to the PSI and II for CO_2_ assimilation. Subsequently, a large amount of light energy dissipated in the form of heat energy in mesophyll cells of senescing leaves. This phenomenon was supported by the result of a significant structural deformation of chloroplast, de-stacking of thylakoid and stromal lamellae in the senescent leaves under LN condition and *psf* mutant (Fig. 1), Therefore, a significant decrease in photosynthesis activities for rice was a necessary result of the decreased harvesting and utilization efficiency of light energy in senescent flag leaves. Hence, these reflected the NTE factor that prevents Chl degradation and N remobilization by high N supply during grain filling period.

Rice yield primarily depends on assimilate translocation from source leaves to developing grains after anthesis and flag leaf is the primary source for these assimilates, predominantly C and N. Therefore, the senescence of source leaves significantly alters C assimilation and N remobilization [7], consequently, it would be challenging to satisfy the demands of C and N simultaneously to grain during leaf senescence [35]. Previous studies have shown that carbohydrates are involved in the regulation of many important physiological processes of plants during stress such as water stress, hormone imbalance, and drought [1, 9]. Our results showed that N deficiency caused sucrose accumulation in the flag leaves before anthesis, but induced glucose accumulation throughout the sampling period, consistently with the temporal pattern of sugar-related isoform gene expression (Fig. 4). Similarly, Chen et al. [4] reported that carbohydrate accumulation is indispensable for induction of leaf senescence under drought conditions. Furthermore, stimulation and regulation of plants photosynthetic are closely related to sugar transcriptional degree. Li et al. [33] reported that the suppression of *OsFrk2* altered the plant phenotype and resulted in low levels of callose deposition, which restricted the sinking movement of sucrose and significantly elevated soluble sugar levels. In our study, LN suppressed *OsFrk2* after anthesis, which may restrict the fructose accumulation and elevated sucrose level. Therefore, it is possible that N deficiency inhibited the expression of *OsFrk2* and caused phloem disruption, thus leading to an abundance of sucrose in the leaves after heading. Indicating that sucrose signaling pathways and hexokinase-dependent glucose might be inactive in senescing flag leaves. By disparity, assimilate translocation is amplified, once the leaf senescence is initiated in cereal crops [36], and sucrose is a major assimilate remobilized from source leaves to developing organs, particularly grains [8]. In our study, OsFrKs and OsCINs, displayed significantly increasing expression levels under LN, whereas SPS and OsSUTs exhibited slightly decreasing expression levels (Fig. 4), indicating that sucrose synthase and cleavage were enhanced and sucrose synthesis was subjugated by beta-fructofuranosidase SPS and, hence, destabilized during senescence. Explaining that, sucrose accumulation is necessary but not sufficient to induce leaf senescence under N deficiency conditions, but glucose inactivates photosynthetic machineries and triggers leaf senescence in collaboration with other carbohydrate metabolism-related genes.

The ant-oxidative system and ROS play central regulatory roles in senescence, tissue development, and stress response in plants. Under natural conditions, the generating and dismissal of ROS in plant cell components are in equilibrium by virtue of activating the antioxidant enzymes: SOD, CAT, APX, and POD. However, stress conditions (abiotic or biotic) often cause a serious disparity in any plant cell segment because of decreasing ant-oxidative capacity and increasing ROS generation [24]. Leaf senescence was physiologically characterized by the generation of ROS in deteriorating tissues and incapacitated by muting activities of antioxidant enzymes under hostile conditions [37]. In the present study, LN supply generated high O_2_^**•−**^ and H_2_O_2_ concurrently with declined in SOD, CAT, and APX., meanwhile the POD showed strength activity as senescence persist, clearly indicating the weakened capacity of antioxidant activities in scavenging O_2_^**•−**^ and H_2_O_2_ through dismutation reaction in senescent leaves, which led to the accumulation of ROS and acceleration of leaf senescence under N deficiency condition, at the same time, specifying the rule and function of POD in the conversion of superoxide anions to H_2_O_2_, which acts as the substrate for catalase, as similarly reported in early senescence rice by Li et al. [23] and in Maize subjected to high temperature from the study of [38]. Furthermore, increased ROS accumulation in chloroplast might be associated with alteration in the ultrastructure of grana and thylakoids in the senescent leaves [10]; as in this study, the increases in the number of plastoglobuli in *psf* mutant chloroplasts might explain the oxidative stress that disrupted N metabolites during premature senescence and its close association with alteration in the ultrastructure of grana and thylakoids in the senescent leaves as a result of high ROS generation in the chloroplast. Likewise, it appears that the accumulation of ROS may be a pre-requisite for C/N metabolism imbalance and, hence, compressed size of the stromal matrix and increases C/N ratios. Considering the changes in the temporal pattern of leaf senescence, phenotype, intercellular sugar levels, the expression level of essential genes encoding for the reaction center of the photosystems and N assimilation, ultrastructure of chloroplast, and other senescence-related physiological parameters together, we proposed that the drastic enhancement in ROS generation and accumulation was firmly related to N-deficiency among the important causative factors for the induction of premature leaf senescence in collaboration with sugar accumulation and, accordingly, resulted in dismantling of the genetic program of the flag leaf to function resourcefully and principally toward remobilization of metabolites to developing sink tissues. It clearly revealed that the flag leaves defense mechanisms could be stimulated when the N supply is limited, which will, in turn, affect physiological and biochemical components mechanism in rice plant. Thus, the changes in both C and N metabolism, ROS content, and antioxidant activity can be used as biomarkers in understanding the effects of N deficiency on the regulation of physicochemical processes in rice leaf during grain filling.

## Conclusion

The initiation and progression of leaf senescence induced by N deficiency led to the acceleration in the transport of pre-stored N and carbohydrates from the flag leaf tissues during grain filling and significantly increase N reducing rate and NSC contribution, hence reducing the grain yield. The *psf* mutant was more susceptible to N deficiency than its WT in the initiation and progression of leaf senescence. The N supply level has a strong influence on sugar accumulation in the rice leaf, and LN-induced downregulation in senescence-associated genes in the flag leaf was closely associated with the accelerated leaf senescence and shortened leaf longevity. Furthermore, the *psf* mutant had relatively weaker N assimilation and stronger C accumulation compared with WT. Under N deficiency conditions, the senescence-related ROS generation and production might be explained as a result of the weak capacity for ROS detoxification triggered by the decrease in CAT, SOD, and APX activities in collaboration with the C/N ratio accumulation. Thus, our findings provide helpful information for plant senescence regulation and improving rice production by appropriate N supply.

## Supplementary Information

**Additional file 1: Table S1.** The sequence of primers for ACTIN and genes used for real-time quantitative PCR. **Table S2.** Differences in grain yield traits, and NSC contribution in the flag leaves of the Wildtype and psf mutant under different nitrogen treatments.

## Data Availability

All data generated or analyzed during this study are included in this published article [and its supplementary information files]. The plant seeds used in this study have been deposited in the seed bank managed by the College of Agriculture and Biotechnology, Zhejiang University, Hangzhou, China, and Jiangsu Collaborative Innovation Center for Modern Crop Production (JCICMCP), Nanjing, China. The generated seeds are available from Prof. Cheng Fangmin of ZJU on practical request with applicable research material transfer agreement and comply with proper national and international regulation.

## Abbreviations

ABA: Abscisic acid
APX: Ascorbate peroxidase
C: Carbohydrate
CAT: Catalase
Chl: Chlorophyll content
C/N: carbon and nitrogen ratio
cDNA: Complementary
DAB: 3,3-diaminobenzidine
POD: Peroxidase
*psf*: Premature senescence flag leaf
H_2_O_2_: Hydrogen peroxide
LN: Low N
N: Nitrogen
NSC: Non-structural carbohydrate
NUE: Nitrogen use efficiency
Pn: Photosynthesis rate
R1/R2: Relative conductivity
ROS: Reactive oxygen species
O^2•−^: Superoxide radical
SOD: Superoxide dismutase
WT: Wild type
