# Supplementary material for: Nitrogen deficiency regulates premature senescence by modulating flag leaf function, ROS homeostasis, and intercellular sugar concentration in rice during grain filling
Source: J Genet Eng Biotechnol. 2021 Nov 23;19:177. doi: 10.1186/s43141-021-00275-3 (PMC8611147; doi:10.1186/s43141-021-00275-3)
Supplement: Supplementary file 1 — Additional file 1: Table S1. The sequence of primers for ACTIN and genes used for real-time quantitative PCR. Table S2. Differences in grain yield traits, and NSC contribution in the flag leaves of the Wildtype and psf mutant under different nitrogen treatments. [file 43141_2021_275_MOESM1_ESM.docx]

Table S1. The sequence of primers for ACTIN and genes used for real-time quantitative PCR

| Gene | Accession No. | Forward primer (5’ → 3’) | Reverse primer (5’ → 3’) | Product size |
| --- | --- | --- | --- | --- |
| Actin | X16280 | CAGCACATTCCAGCAGATGT | TAGGCCGGTTGAAAACTTTG | 146 |
| GS1 | XM_015770616 | TCGTCGTCTCATTTGACCCC | TGGCGGACTTGATGATCTCG | 173 |
| GS2 | XM_015779837 | CAAACGTAACAGGGCTGCAC | CCAGCCAAGAGGCCAGTTTA |  |
| Cab | 4346803 | TGGCAGGACATCAAGAACCC | GCTCCTTCTCCTTGGCCTC | 145 |
| PsbA | 3131409 | ATCTGTAGTTGATAGCCAAGGTCG | TAGGTCTAGAGGGAAGTTGTGAGC | 118 |
| PsbB | 3131410 | ACGGTGGAGTTCTATGGTGG | CCCTTGGACTGCTGCGAAA | 154 |
| PsbC | 3131411 | GGAGCAATGAACCTATTTGAAGTGG | GCCTAAGACTGCGGAGGAAAT | 186 |
| PsbD | 3131412 | AACCGCAGCAGTTTCCACC | CACCAACGAGTAAAATCCCCTT |  |
| SGR | P0515E01.8 | AAGGAGCTCCCCGTGGTTCTGAAGGC | TACCGCCCTTCGTCGTCAGCGTGA |  |
| NAP | Os03g0327800 | GCACCGACAAGCCCATCCACAGC | ATCCGGCACAGCACCCAGTCATCC |  |
| SPS1 | NM_001051745 | GAGACTTCGGAGGACCTTGCA | TGGGAGTGTCTTGCTGTGCC | 85 |
| SPS2 | NM_001052643 | TGATAATGGGTAACCGTGAGGC | CAGAGTGCTTATGATGCTTGGGAT | 135 |
| SPS6 | NM_001064716 | CGTGGTAGAAAGAAGAGGGAGGA | CAGAGCGTGACAACGGAGTGA | 166 |
| SPS8 | NM_001068030 | GGAGATTCCTTGAGGGATGTTC | CCATTTATTGCCTGTGGTAGCC | 195 |
| SPS11 | NM_001074101 | CCAAGCACCACAAGCAGACC | GTTCTTCGTCGCCACTACCG | 152 |
| CIN1 | AY342319 | TACACGGTGGGCATCTACAA | TCCAGCCATACCTTTCTTGG | 236 |
| CIN2 | AY340072 | CTCTGAGGAGCCTGATCGAC | AGGCTCCATTCATCATGACC | 201 |
| CIN4 | AY578161 | CATGTTTGTGCCGGATACTG | TGCCATCACCATCTAACCAA | 218 |
| SuSy1 | AK100334 | TGACTGGTCTGGTTGAGCTG | ACAAAAGCACCCTTGGTGTC | 247 |
| SuSy2 | AK072074 | TCCTGGAGCTGACATGAGTG | AAGACGAGCCATGGAGAAGA | 169 |
| SuSy3 | AK100306 | TTCACAGCTGACCTGATTGC | CATGTCAGCACCAGGAGAGA | 198 |
| SuSy4 | AK102158 | AGCCAATGTGTTGGGCTTAC | GCATGATGTCCCTTTTGCTT | 172 |
| SuSy5 | AK063304 | TCTCAACACCCCATTTGACA | GTGTATTTTGGCAGCCCACT | 241 |
| SuSy6 | AK065549 | CAAGCTGACCGACAACAAGA | TGCATGTTTCAGCAGTGTCA | 236 |
| SUT1 | D87819 | GCCAAGGAAGTTCCATTCAA | TGAGGATCAGTTCCCTTTGG | 242 |
| SUT2 | AB091672 | TGCAAGAGCCTCAATCCTTT | AATGGAAACCATCCAATCCA | 160 |
| SUT3 | OS10g0404500 | ATAAGCCTGTCGGGGCTATT | CCAGTGAGGATGAACGGTCT | 239 |
| SUT4 | AB091673 | GCATGCTGGCTACAGCAATA | AGCAGTCAGCTCAGCAGTCA | 182 |
| SUT5 | AB091674 | AAGGTCTTGCCATTGGTGTC | GTGGTGCCTTTGATGGAGTT | 243 |

Table S2. Differences in grain yield traits, and NSC contribution in the flag leaves of the Wildtype and psf mutant under different nitrogen treatments

| Cultivar | Treatment | Available panicle per plant | Number of grains per panicle | Number of filling grains per panicle | 1000 seed weight | Seed setting rate (%) | Grain yield per plant (g) | N reducing rate | NSC contribution to grain (%) |
| --- | --- | --- | --- | --- | --- | --- | --- | --- | --- |
| WT | LN | 9.92c | 109.67c | 42.33c | 20.69b | 38.60c | 0.91c | 57.76a | 1.353a |
|  | MN | 10.31b | 152.01b | 119.82a | 22.38a | 78.82 a | 2.76ab | 45.96b | 0.227b |
|  | HN | 12.34a | 163.65a | 106.64b | 20.42b | 62.11b | 2.56b | 37.84c | 0.135b |
|  | Mean | 10.86A | 141.78A | 87.93A | 21.50A | 59.84A | 2.08A | 47.19B | 0.371B |
| *psf* | LN | 7.96c | 88.73b | 9.25c | 18.75b | 10.42c | 0.14c | 59.90a | 8.296a |
|  | MN | 9.35b | 132.67a | 40.81b | 19.86a | 30.76b | 0.76b | 52.75b | 1.530b |
|  | HN | 10.33a | 134.16a | 75.67a | 20.05a | 56.40a | 1.57a | 50.35b | 0.371c |
|  | Mean | 9.21B | 118.52B | 41.99B | 19.55B | 32.53B | 0.82B | 54.33A | 1.442A |

Different letters within column indicate a significant difference between nitrogen treatments and mean value significant difference between the cultivars under all the treatments at p ≤ 0.05 level

NSC contribution = [NSCs in leaf at the heading stage - NSCs residue of the leaf at the harvest stage]/weight of filling grains per panicle x 100

N reducing rate = [leaf N content at heading - leaf N content at harvest stage]/ leaf N content at heading x 100
